# Bridging the Digital Divide: Factors Affecting Asynchronous and Synchronous Telehealth Use

**DOI:** 10.1093/geroni/igaf122.1143

**Published:** 2025-12-31

**Authors:** Su-I Hou, Jae Park

**Affiliations:** University of Central Florida, Orlando, Florida, United States; University of Central Florida, Orlando, Florida, United States

## Abstract

**Introduction:**

Telehealth has transformed healthcare, offering synchronous (real-time video/phone) and asynchronous (patient portal) options. This study examines factors influencing telehealth adoption, particularly age-related differences.

**Methods:**

Data from the 2022 Health Information National Trends Survey (HINTS) 6 were analyzed, assessing asynchronous (patient portals) and synchronous (telehealth visits) use. Key variables included demographics, tech-savviness, and healthcare behaviors, with age groups categorized as young (18-49), middle-aged (50-64), and old (65+). Stepwise regression models identified significant factors.

**Results:**

Among 5,873 participants (35.3% young, 29.0% middle-aged, 35.7% old), 61.6% used asynchronous and 41.9% used synchronous telehealth. Males were less likely to use telehealth (OR = 0.66, p < 0.001), but older males favored synchronous use (OR = 1.55, p = 0.002). Hispanics preferred synchronous (OR = 1.42, p < 0.001) but used asynchronous less (β = -0.35, p < 0.001). Rural/smaller urban areas had lower use (OR range: 0.63–0.78, p < 0.001). Tech-savviness was linked to telehealth use, especially asynchronous (e.g., internet use: β = 0.53, p < 0.001). Online survey use was associated with asynchronous telehealth, particularly for middle-aged (β = 0.20, p = 0.024) and older adults (β = 0.24, p = 0.006). Frequent healthcare users were more likely to use both (OR = 1.28, p < 0.001; β = 0.28, p < 0.001), but older adults were less likely to use asynchronous telehealth (β = -1.21, p < 0.001).

**Discussion:**

Age, gender, ethnicity, location, and tech-savviness impact telehealth adoption. Addressing disparities requires broadband expansion, digital literacy programs, and age-friendly telehealth models.

